# Study on the Driving Factors of Plankton Community and Water Health Under the Terrain Barrier: A Case Study of Xinjiang

**DOI:** 10.3390/biology15030238

**Published:** 2026-01-27

**Authors:** Long Yun, Changcai Liu, Xuelian Qiu, Fangze Zi, Wenxia Cai, Liting Yang, Yong Song, Shengao Chen

**Affiliations:** 1 State Key Laboratory Incubation Base for Conservation and Utilization of Bio-Resource in Tarim Basin, Tarim Research Center of Rare Fishes, College of Life Sciences and Technology, Tarim University, Alar 843300, China; 10757232147@stumail.taru.edu.cn (L.Y.); 107572025416@stumail.taru.edu.cn (C.L.); 10757231124@stumail.taru.edu.cn (X.Q.); or 2024400189@buct.edu.cn (F.Z.); 10757252199@stumail.taru.edu.cn (W.C.); 10757231127@stumail.taru.edu.cn (L.Y.); 2 College of Material Science and Engineering, Beijing University of Chemical Technology, Beijing 100029, China

**Keywords:** arid regions, zooplankton functional group, regional barrier, water environment

## Abstract

This study investigated the distribution patterns of zooplankton species composition and functional groups, their correlations with aquatic environmental factors, and the mechanisms underlying community stability under the influence of regional barriers in arid areas of Xinjiang, China. The aim was to elucidate the ecological processes driving zooplankton communities in artificial aquatic ecosystems in Central Asia. A systematic survey was conducted on water environmental parameters and zooplankton community structures across 10 artificial water bodies, including the southern foot of the Altai Mountains and both northern and southern slopes of the Tianshan Mountains. The survey encompassed physical and nutrient indicators, and the results revealed significant spatial variation among water bodies across regions. Artificial water bodies in the southern Altai Mountains and northern Tianshan Mountains exhibited substantial fluctuations in temperature, dissolved oxygen (DO), total nitrogen (TN), and total phosphorus (TP). In contrast, water bodies in the southern Tianshan Mountains showed less variation in nutrient indicators. Zooplankton identification results indicated marked differences in zooplankton communities across regions, which were further confirmed by cluster analysis and non-metric multidimensional scaling (NMDS). A total of 19 dominant zooplankton species were identified across the three basins, classified into 6 functional groups. The composition of zooplankton functional groups also varied considerably, which may be closely associated with significant fluctuations in nutrient indicators of aquatic environmental factors across regional barriers. Additionally, there were specific differences in zooplankton diversity among the three basins: the SA region ranged from α-mesosaprobic to polysaprobic and β-mesosaprobic; the NT region was classified as β-mesosaprobic; and the ST region ranged between β-mesosaprobic and lightly polluted. These results may be attributed to differences in regional barriers and glacial meltwater conditions. Canonical Correspondence Analysis (CCA) showed that environmental factors collectively explained 71.1% of the variation in species distribution. Exploring the zooplankton species composition and their relationships with aquatic environmental factors under different regional barriers provides a scientific basis for regional water resource management and environmental protection.

## Simple Summary

To elucidate the driving mechanisms of zooplankton communities in Central Asian artificial aquatic ecosystems, this study surveyed water environmental parameters and zooplankton communities in 10 artificial water bodies at the southern Altai foot and Tianshan’s northern/southern slopes. Results revealed significant spatial variations: water bodies in the southern Altai and northern Tianshan showed substantial fluctuations in temperature, dissolved oxygen (DO), total nitrogen (TN), and total phosphorus (TP), whereas southern Tianshan waters showed more minor nutrient indicator variations. Marked regional differences in zooplankton communities were confirmed by cluster analysis and non-metric multidimensional scaling (NMDS). Across three basins, 19 dominant species were identified, grouped into 6 functional groups with distinct compositional differences—closely linked to nutrient fluctuations induced by regional barriers. Diversity varied by region: SA ranged from α-mesosaprobic to polysaprobic; NT was β-mesosaprobic; and ST ranged from β-mesosaprobic to lightly polluted. These differences were attributed to regional barriers and variations in glacial meltwater. Canonical Correspondence Analysis (CCA) showed that environmental factors collectively explained 71.1% of the variation in species distribution. This study demonstrates that regional barriers regulate zooplankton communities by altering aquatic conditions, with glacial meltwater exacerbating these differences, providing a scientific basis for the management and protection of artificial water bodies in arid areas.

## 1. Introduction

In aquatic ecosystems, zooplankton are a group of generally small-sized organisms with relatively limited active migration capabilities. Their food sources mainly consist of microorganisms such as bacteria and phytoplankton, and they also serve as crucial forage resources for aquatic organisms such as filter-feeding fish and shellfish. Functioning as a vital link connecting lower and higher trophic levels in the food chain [1], zooplankton play an indispensable role in sustaining ecological processes within aquatic ecosystems, including material cycling, energy flow, and information transmission [2]. In recent years, studies have shown that zooplankton respond rapidly to environmental changes, particularly to pollutants. They can accumulate and transfer contaminants, and changes in their community succession and diversity can effectively reflect the characteristics and trends of aquatic environmental quality [3,4]. Consequently, zooplankton are frequently employed as excellent bioindicators in environmental monitoring and assessment [5]. In large freshwater ecosystems such as lakes, the species composition, ratios, and quantitative characteristics of zooplankton can respond quickly to changes in the surrounding freshwater environment, rendering them of significant application value in fields including limnological research, water eutrophication monitoring, and freshwater ecological protection [6].

In ecological investigations, topographic barriers are typically characterized as natural landform-related elements within the geographical setting, encompassing physical features like mountains, rivers, lakes, and analogous natural formations [7]. These natural barriers can significantly regulate the geographical distribution, migration behavior, and population dynamic processes of organisms [8]: on the one hand, they spatially separate ecosystems in different regions, enabling biological communities on both sides of the barriers to evolve gradually in relatively isolated environments, thereby differentiating into unique subspecies or even new species [9]; on the other hand, this type of spatial separation functions as a natural impediment to the dispersal pathways of migratory organisms, leading populations in each segregated area to develop distinct evolutionary pathways. This phenomenon not only boosts local species diversity but also exerts a profound influence on the overall population structure and geographical distribution patterns of species [10]. In addition, topographic barriers are likely to impose heterogeneous natural selection pressures on organisms through modifying local environmental conditions. Such selection forces can exert dual impacts either positive facilitation or negative restraint on biodiversity across varying ecological scenarios, ultimately prompting species to evolve adaptive traits at the phenotypic, physiological, or genetic levels [11]. Topographic barriers exert a substantial regulatory effect on the distribution patterns of zooplankton, and the core mechanism lies in their indirect impact on zooplankton’s migratory behaviors, survival tactics, and community configurations by altering critical ecological factors including hydrodynamic regimes, light conditions, temperature gradients, and material cycling processes [12]. In ecological systems, topographic components like mountain ranges and islands are capable of substantially disrupting the vertical and horizontal circulation of water bodies, hindering the regular water exchange between different water columns. This kind of disruption gives rise to the formation of heterogeneous flow regimes on both sides of the barriers. Such hydrodynamic discrepancies directly impact the dispersal efficiency and distribution scope of zooplankton: stagnant water zones often promote the aggregation of zooplankton, whereas high-velocity flow zones may limit their capacity for active migration [13]. In rivers or lakes flowing through mountainous regions, topographic undulations drive pronounced temperature gradients, with water temperatures in high-altitude areas being significantly lower than those in low-altitude sections [14]. Given that zooplankton metabolic rates, reproductive cycles, and community composition are highly sensitive to temperature, these topographically mediated temperature differences indirectly reshape their spatiotemporal distribution patterns [15]. Additionally, mountain ranges modulate the transport of dissolved nutrients, trace elements, and suspended particulate matter in river systems by modifying the pathways of surface runoff [16,17,18].

Xinjiang, China’s largest administrative region, is situated in the heart of Central Asia, where a geographically intricate landscape unfolds, encompassing vast desert plains, expansive grasslands, towering mountain ranges, and numerous lakes. The Altai and Tianshan Mountains stand as two of the region’s most defining geographical landmarks. Nestled in northern Xinjiang, the Altai Mountains form natural borders with Mongolia and Russia, while also spanning across Central Asia. Comprising a series of interconnected mountain ridges that traverse China, Mongolia, and Russia, this range boasts a distinctive topography marked by lofty peaks, deep gorges, and scattered lakes [19]. The climatic and hydrological dynamics of the Altai Mountains play a crucial role in shaping the environmental conditions of northern Xinjiang, resulting in distinct climatic and ecological patterns on either side of the range. Nestled in central Xinjiang, the Tianshan Mountains stand as one of the world’s longest mountain systems, stretching east–west and effectively dividing the region into northern and southern halves. Its towering peaks and deep canyons have contributed to the diverse topographical landscape across Xinjiang, each feature carving out unique microclimates and habitats [20].

Xinjiang, China, is among the regions globally grappling with the most critical water scarcity, yet it sustains an impressive level of water resource utilization efficiency. Despite the Tarim River Basin’s abundant groundwater resources, their spatial distribution is highly uneven, and the water quality is generally characterized by high mineralization, rendering exploitation and utilization relatively challenging. Against this backdrop, the construction of artificial lakes and reservoirs often requires careful consideration of topographic, water resource, and environmental factors, which may affect surrounding ecosystems and socioeconomics [21]. Zooplankton communities in artificial water bodies are closely associated with topographic barriers [22]. Topographic obstacles such as hills and mountains can create environmental isolation within water bodies, alter hydrodynamics, and create diverse habitats [23]. These topographic barriers induce specific circulation patterns in water bodies, thereby affecting nutrient transport and distribution and ultimately influencing zooplankton growth and distribution. Due to differences in environmental conditions across regions, topographic barriers can increase the diversity and abundance of zooplankton communities [24].

In recent years, research on zooplankton communities in Chinese reservoirs has received increasing attention. Lin et al. [25] surveyed 18 tropical reservoirs in South China, identifying 98 zooplankton species. They found that lentic reservoirs with short retention times exhibited high species richness but low abundance, whereas reservoirs with long retention times showed the opposite pattern. Hu et al. [26] focused on reservoirs in Xinjiang, revealing that water temperature, dissolved DO, nitrogen, phosphorus, and silicon are key physical and chemical factors regulating zooplankton distribution. They also proposed that rotifer density could serve as a bioindicator for eutrophication in deep reservoirs. Liu et al. [27] studied the body size structure of zooplankton in the Xiwan section of the Three Gorges Reservoir, finding that nutrients indirectly affect zooplankton body size diversity by regulating phytoplankton chlorophyll a, while increased water temperature exerts a significant negative impact on body size diversity. These studies have collectively deepened our understanding of zooplankton communities in Chinese reservoirs, covering community composition, environmental drivers, variations in body-size structure, and the application of monitoring technologies. However, research on zooplankton species composition and the driving factors of diversity changes under regional barriers in alpine regions remains scarce. Through community and correlation analyses, this study found significant differences in zooplankton diversity across water bodies—regions with more complex topographic features exhibited higher zooplankton diversity. The results will lay a scientific foundation for the systematic monitoring of zooplankton diversity in extensive water environment assessment and for the protection and development of fishery resources.

## 2. Materials and Methods

### 2.1. Study Area

The Altai and Tianshan Mountains stand as two of Xinjiang’s most defining geographical landmarks. Nestled in the northern reaches of the region, the Altai Mountains form natural boundaries with Mongolia and Russia, and they also lie within the broader Central Asian geographic context. Comprising a series of interconnected transverse mountain ridges that span China, Mongolia, and Russia, this mountain system, along with its towering peaks, deep valleys, and scattered lakes, collectively creates a distinctive topographical landscape.

**Figure 1:**
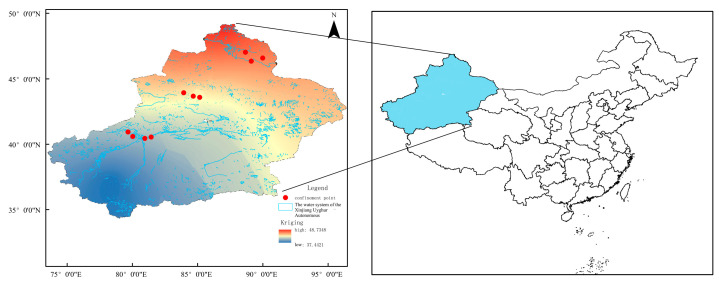
Sampling Map of zooplankton in Ten Artificial Water Bodies in Xinjiang.

Located in central Xinjiang, the Tianshan Mountains rank among the world’s longest mountain ranges. Stretching east–west across the region, they effectively split Xinjiang into its northern and southern halves. Their imposing peaks and dramatic canyons have played a pivotal role in shaping the diverse topography across the entire region. Their peaks and valleys have shaped diverse topographic features across Xinjiang. Given the unique geographical environment of Xinjiang, we selected 10 artificial water bodies as zooplankton sampling sites at the southern foot of the Altai Mountains and both northern and southern slopes of the Tianshan Mountains from 2022 to 2023 (Figure 1). Four artificial water bodies south of the Tianshan Mountains were designated as Xinjingzi Reservoir (XJZ), Shangyou Reservoir (SY), Duolang Reservoir (DL) and Shengli Reservoir (SL). Three artificial water bodies north of the Tianshan Mountains included Liugou Reservoir (LG), Daquangou Reservoir (DQG) and Moguhu Reservoir (MGH). Another three artificial water bodies south of the Altay Mountains were Akedala Reservoir (AKDL), Nangan Canal (NGQ) and Kezisai Reservoir (KZS), as presented in Table 1. For each of the 10 artificial water bodies, five sampling sites were established, corresponding to the water inlet, water outlet, deep-water area, shallow-water area and littoral zone, respectively. Sampling campaigns were conducted on four occasions, specifically in September and November 2022, as well as March and June 2023. All data utilized in this study were the mean values derived from the four sampling events at each individual sampling site.

### 2.2. Sample Collection and Determination Methods

#### 2.2.1. Physicochemical Indices of the Water

Water temperature (WT), pH, dissolved oxygen (DO), total dissolved solids (TDS), conductivity (C), salinity (SAL), and oxidation–reduction potential (ORP) data were measured on-site using a water quality detector (the ATI PQ45 multiparameter water quality analyzer from Analytical Technology, Inc., Collegeville, PA, USA). Transparency was measured using a Secchi disk (JCT-8S, Qingdao Juchuang Environmental Protection Group Co., Ltd., Qingdao, China). Indices such as latitude, longitude, and altitude were recorded using a Global Positioning System (GPS) unit (LS-GPS-639, Qingdao Lvsheng Biotechnology Co., Ltd., Qingdao, China). Water chemical parameters (TN, TP) were determined in the laboratory according to the guidelines in “Methods for the Examination of Water and Wastewater (Fourth Edition)” [28]. The sampling time and location data correspond to the biological data.

#### 2.2.2. Zooplankton

Zooplankton samples were collected using a No. 13 plankton net with a mesh size of 0.112 mm. Sampling was conducted in transparent plastic reagent bottles with a wide opening, each with a capacity of 50 mL. In addition to a pipette with a rubber bulb (internal diameter of 2 mm), a 0.1 mL counting chamber, and a 20 mm × 20 mm coverslip, Lugol’s solution and a 4% formaldehyde solution were used to fix and preserve the collected samples. Subsequent observation of the zooplankton was performed using a Leica BME microscope (Leica Microsystems (Shanghai) Trading Co., Shanghai, China). The classification and identification of zooplankton are based on Fauna Sinica—Arthropoda: Crustacea: Freshwater Copepoda; Fauna Sinica—Arthropoda: Crustacea: Freshwater Cladocera; and the Monograph of the Chinese Freshwater Rotifers [29,30,31].

The enumeration of zooplankton was performed using the row-grid technique within a specialized counting chamber. Initially, a 30 mL volume of the concentrated water sample was vigorously agitated. Subsequently, a precise 0.1 mL aliquot was transferred from this sample to a 0.1 mL counting chamber utilizing a calibrated pipette. A standard 22 mm × 22 mm cover glass was then carefully placed over the Hausser Scientific HS3850 Palmer Nanoplankton Counting Chamber (935 Horsham Road, Suite C, Horsham, PA, USA) (0.1 mL) to ensure proper sample distribution. Under a high-power microscope, a systematic count was conducted on 30 individual small grids, specifically selected from the 2nd, 5th, and 8th rows of the counting chamber (note: the entire chamber consists of 10 rows, each containing 10 grids, totaling 100 counting units). These counts were further categorized by species. Finally, the derived count data were employed to calculate the total zooplankton abundance per liter of the original water sample using the following formula [32,33,34]:

$$N=\frac{V\cdot P}{W\cdot C}$$

In the formula, the letters are expressed as follows:

*P*: the number of zooplankton observed under the microscope (the average of two slides);

*C*: the volume of the counting chamber (mL);

*V*: the volume of the 1 L water sample after precipitation and concentration (mL);

*W*: the volume of the sampled water (L).

The zooplankton biomass was determined by wet weight using a No.13 net.

### 2.3. Division of Zooplankton Functional Groups

In freshwater ecosystems, zooplankton are classified into nine functional groups based on body size, feeding habits, reproductive mode, life cycle, and escape strategies: Rotifer Filter-feeders (RF), Rotifer Carnivores (RC), Rotifer Herbivores (RP), Small Zooplankton Filter-feeders (SZF), Small Zooplankton Carnivores (SCC), Medium-sized Zooplankton Filter-feeders (MCF), Medium-sized Zooplankton Carnivores (MCC), Large Zooplankton Filter-feeders (LCF), and Large Zooplankton Carnivores (LCC) (Table 2) [35]. In this study, functional communities composed of 19 dominant species (*Y* > 0.02) were selected as the research objects, as these results more intuitively reflect differences in functional communities under varying spatiotemporal conditions. The dominant species were categorized into six zooplankton functional groups: Rotifer Filter-feeders (RF), Small Copepods and Cladocera Filter-feeders (SCF), Small Zooplankton Carnivores (SCC), Medium-sized Zooplankton Filter-feeders (MCF), Rotifer Carnivores (RC), and Medium-sized Zooplankton Carnivores (MCC).

### 2.4. Data Processing and Analysis

#### 2.4.1. Calculation of Dominant Species

The calculation formula for the dominant species of zooplankton is as follows [37]:

$$Y=(\frac{n_{i}}{N})f_{i}$$

In the formula,

*n_i_*: The variable represents the proportion of individuals belonging to the i-th species relative to the overall number of individuals;

*N*: The total number;

*f_i_*: The frequency of occurrence of the *i*-th species. When *Y* > 0.02, this species is regarded as a dominant species.

#### 2.4.2. Biodiversity

Diversity index of zooplankton (Shannon–Wiener, *H*′).

Shannon–Wiener diversity index (*H*′) calculation formula:

$$H^{\prime }=-\sum p_{i}\times {log}_{2}p_{i}$$

Richness index of zooplankton (Margalef, *d*).

Margalef richness index (*d*) calculation formula:

$$d=(S-1)/{log}_{2}N$$

Evenness index of zooplankton (Pielou, *J*′).

Pielou evenness index (*J*′) calculation formula:

$$J^{\prime }=H^{\prime }/{log}_{2}S$$

Zooplankton diversity index (Simpson, *D*).

Simpson diversity index (*D*) calculation formula:

$$D=1-\sum {Pi}^{2}$$

*S*: the number of species in the community;

*N*: the total number of individuals observed in the quadrat;

*Pi*: the proportion of the number of individuals of the *i*-th species at this station out of the total number of individuals.

In the above analysis, PRIMER 5.2.8 was used to calculate the Margalef species richness index (*d*), the Shannon–Wiener diversity index (*H*′), the Simpson dominance index (*D*), and the Pielou evenness index (*J*′) to measure and analyze the biodiversity of the zooplankton community [38,39,40,41].

#### 2.4.3. Canonical Correspondence Analysis (CCA)

Detrended Correspondence Analysis (DCA) was executed via Canoco 5 software (Windows 4.5 version). The analytical outcome indicated that the standard deviation (SD) value surpassed 3. Consequently, Canonical Correspondence Analysis (CCA) was subsequently conducted by integrating species data with corresponding environmental variables, aiming to explore the impacts of multiple environmental factors on zooplankton [42].

#### 2.4.4. Bray–Curtis Similarity Analysis

The Bray–Curtis similarity coefficient was computed using the biomass data of zooplankton functional groups. SPSS Statistics 27 was then employed to perform Multidimensional Scaling (MDS) analysis, which aimed to quantify the dissimilarities in zooplankton functional group compositions across different seasons and sampling sites [43].

#### 2.4.5. NMDS + PERMANOVA Analysis

To evaluate the similarities and dissimilarities in the composition of zooplankton functional groups, the NMDS (Non-metric Multidimensional Scaling) algorithm was employed. Complementarily, PERMANOVA (Adonis), a nonparametric multivariate analysis of variance based on Bray–Curtis distances, was utilized to test and validate the statistical significance of these differences. For revealing the spatial distribution patterns of zooplankton functional groups across sampling sites, the same NMDS algorithm was applied to reduce the dimensionality of community compositional data and visualize the degree of similarity between samples. The significance of differences between functional groups was further assessed using PERMANOVA with 999 permutations. Additionally, to ensure the reliability of the results, the stability of stress values was evaluated through 200 repeated runs of the analysis [44].

#### 2.4.6. Correlation Analysis and Mantel Test Correlation Test

After log10-transforming zooplankton functional group communities to enhance their normality, Pearson correlation analysis was performed using the average linkage clustering method and Euclidean distance metric to explore the relationships between distinct zooplankton functional group communities and environmental variables [43]. One-way ANOVA in IBM SPSS Statistics 21 was employed to analyze temporal variations in environmental factors, with *p* < 0.05 considered statistically significant. To assess the significance of the associations between environmental factors and zooplankton functional group communities, a Mantel test was applied, and the Bonferroni method was used for *p*-value adjustment. The Mantel test heatmap was generated using the ‘vegan’ package in R 4.3.1. Variance Inflation Factors (VIF) were calculated to evaluate collinearity among environmental variables. All these analyses were conducted with homogeneity tests and normal distribution corrections.

## 3. Results

### 3.1. Distribution Characteristics and Correlation of Water Environment Factors

**Figure 2:**
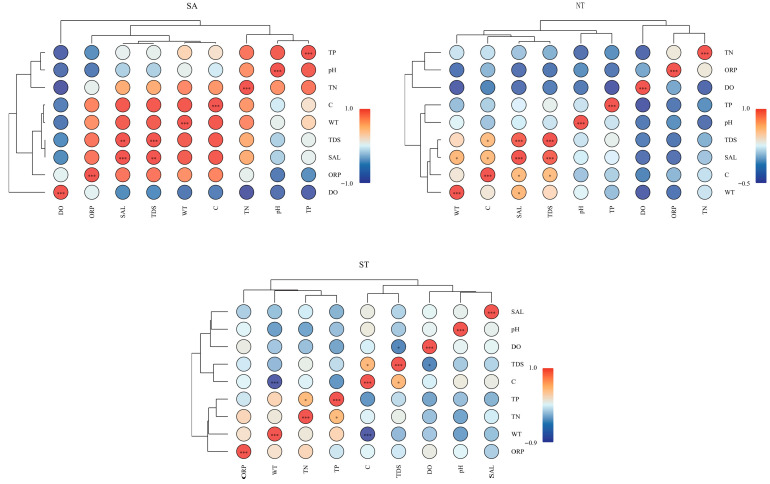
Correlation analysis of water environment factors in three regions. *p < 0.05, ** *p* < 0.01, *** *p* < 0.001.

Results of this study on artificial aquatic environments in Central Asia demonstrate that water environmental indicators (physical parameters: WT, ORP, pH, DO, C, SAL, TDS) and nutrient indicators (TN, TP) exhibit varying degrees of spatial variability. In the southern Altai Mountains, a physical parameter analysis revealed a strong correlation between SAL and TDS (*p* < 0.01). In the northern Tianshan Mountains, C was highly correlated with TDS and SAL (*p* < 0.05), while WT showed a strong correlation with SAL (*p* < 0.001). In the southern Tianshan Mountains, the physical parameters C and TDS showed significant positive correlations (*p* < 0.05) and significant negative correlations with WT (*p* < 0.001), and TN was significantly correlated with TP (*p* < 0.01) (Figure 2).

**Figure 3:**
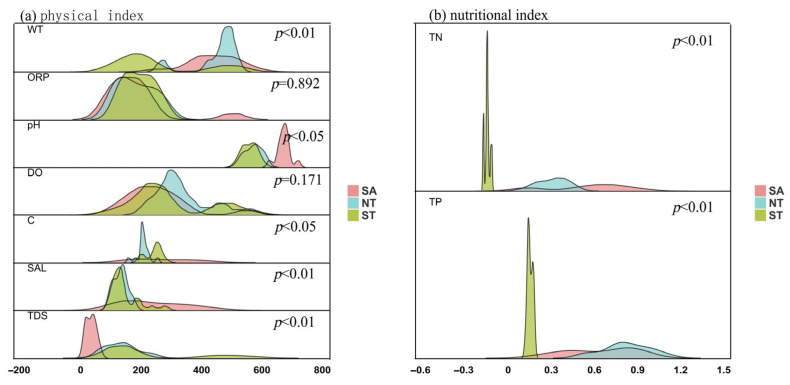
Differences in Environmental Physics and Nutrient Indexes in Three Areas of Sewage Discharge.

As illustrated in Figure 3a, the water temperature (WT) in the southern Tianshan Mountains (ST) was slightly lower than that in the other two regions; the oxidation-reduction potential (ORP) in the southern Altai Mountains (SA) was considerably higher than in the other regions; the pH value in SA was higher than in the northern Tianshan Mountains (NT) and ST; the dissolved oxygen (DO) levels overlapped substantially among the three regions; both conductivity (C) and salinity (SAL) were higher in SA; and the total dissolved solids (TDS) in SA were significantly lower than in the other two regions.

Water quality analysis across the three regions revealed that, except for ORP and DO, WT, C, SAL, and TDS exhibited significant spatial variations (*p* < 0.01), while pH also showed a significant spatial difference (*p* < 0.05).

Figure 3b shows the spatial variation in nutrient factors in the aquatic environment. Both total nitrogen (TN) and total phosphorus (TP) differed significantly among the three regions (*p* < 0.01). TN levels in SA and NT were higher than in ST but showed greater variation, whereas TN concentrations in ST remained stable. Similarly, TP levels in SA and NT were higher than in ST, with large fluctuations and poor stability. In contrast, both TN and TP in ST remained within a stable range with minimal variations.

### 3.2. Differences in Zooplankton Community Structure in Different Geographical Regions

**Figure 4:**
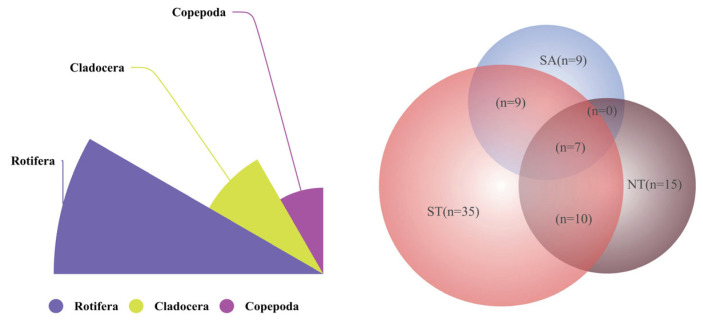
Species composition of zooplankton and Venn diagram of three regions.

A total of 85 zooplankton species were identified across 10 artificial water bodies in three regions (SA, NT, and ST) in this study (Figure 4), including 47 rotifer species (accounting for 55.29%), 15 copepod species (17.65%), and 23 cladoceran species (27.06%). Significant differences in species composition were observed among the three regions, with only seven species shared across all regions. Specifically, ST and SA shared nine species, while ST and NT shared 10. Notably, no shared species were found between SA and NT, indicating substantial dissimilarity in species composition among the three regions.

**Figure 5:**
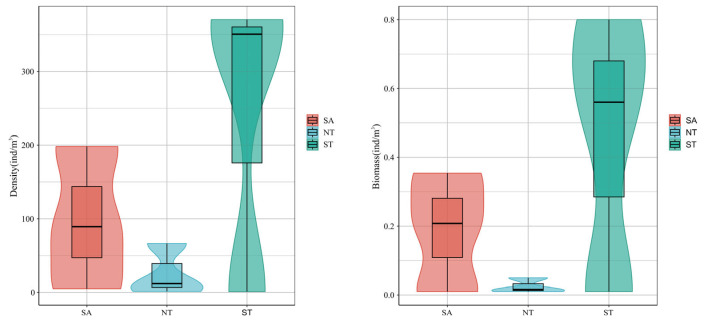
Biological biomass and density of zooplankton in three regions.

Variations in zooplankton abundance and biomass are illustrated in Figure 5. Regarding abundance, ST exhibited the highest values, followed by NT, while SA showed the lowest (see Figure 4). This pattern is likely associated with local physical and nutrient indicators: ST featured minimal environmental fluctuations and relatively stable conditions, whereas significant variations in nutrient and physical parameters in NT and SA may have contributed to their lower zooplankton abundance. Biomass variations across the three regions (presented in the Figure 4) followed a consistent trend with abundance: ST > NT > SA. Notably, the relative biomass in NT was further reduced compared to its abundance, indicating that small-sized rotifers dominated the zooplankton community in NT.

**Figure 6:**
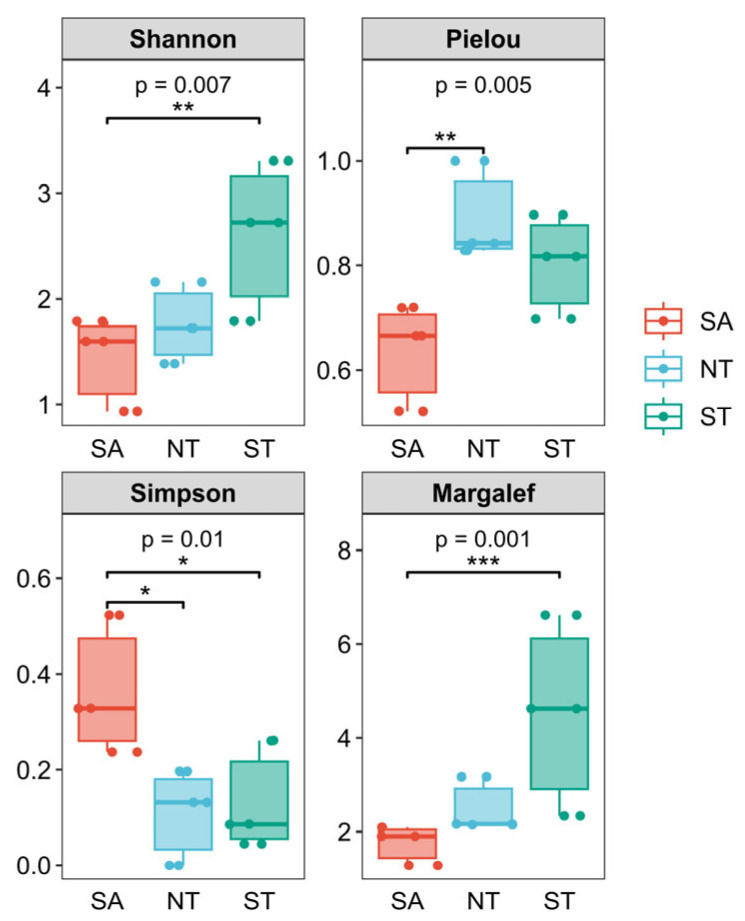
Plankton diversity index in three regions. * *p* < 0.05, ** *p* < 0.01, *** *p* < 0.001.

The zooplankton diversity indices across the three regions are illustrated in Figure 6. The Shannon diversity index indicated that ST had the highest value, followed by NT, with SA the lowest; the difference between SA and ST was highly significant (*p* < 0.01). For the Pielou evenness index, NT exhibited the highest value, followed by ST, while SA was the lowest, with a highly significant difference between SA and NT (*p* < 0.01). The Simpson diversity index showed that SA had the highest value, followed by ST, with NT being the lowest. Significant differences were observed between SA and NT (*p* < 0.05), as well as between SA and ST (*p* < 0.05). Regarding the Margalef richness index, ST displayed the highest value, followed by NT, with SA the lowest; a highly significant difference was detected between ST and SA (*p* < 0.001). Overall, the diversity and richness of zooplankton in ST were substantially higher than in the other two regions, which may be attributed to the relative stability of nutrient indicators in ST.

Analysis of the classified zooplankton community diversity indices and richness indices revealed significant differences among the three regions. Specifically, the diversity indices ranged from 0.93 to 3.30, while the richness indices ranged from 1.27 to 6.61. In the SA region, the diversity index was categorized as “Poor,” and the richness index was rated “Bad.” In the NT region, both the diversity and richness indices fell into the “Moderate” category. The ST region exhibited a “Good” diversity index and a “High” richness index as shown in Table 3.

### 3.3. Species Composition and Water Environment Analysis of Zooplankton Functional Groups in Different Regions

**Figure 7:**
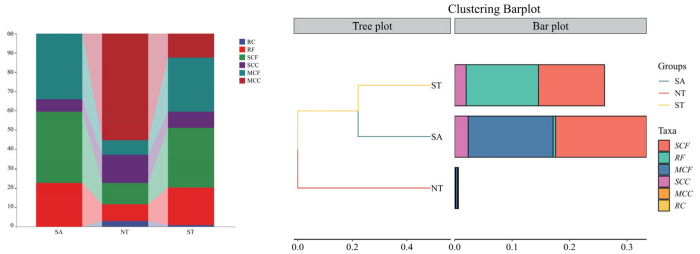
Species composition and similarity of zooplankton functional groups in three regions.

The dominant zooplankton species and their dominance values across the three regions are illustrated in Figure 7. A total of 19 dominant species were identified across the three regions, with only one species (*Cyclopoida larva*) shared among all three regions, indicating substantial differences in dominant species composition (Table 4).

In this study, functional communities composed of 19 dominant species (dominance index *Y* > 0.02) were selected as the research objects, as these results more intuitively reflect differences in functional communities under varying spatiotemporal conditions. The dominant species were classified into 6 zooplankton functional groups: Rotifer Filter-feeders (RF), Small Copepods and Cladocera Filter-feeders (SCF), Small Zooplankton Carnivores (SCC), Medium-sized Zooplankton Filter-feeders (MCF), Rotifer Carnivores (RC) and Medium-sized Zooplankton Carnivores (MCC).

The biomass composition and similarity of dominant functional groups across the three regions are illustrated in the figures. Significant differences were observed in the proportional distribution of functional groups across the three regions, with no significant similarity between them. In the SA region, the functional group composition was dominated by Medium-sized Zooplankton Filter-feeders (MCF), Small Copepods and Cladocera Filter-feeders (SCF), and Rotifer Filter-feeders (RF), accounting for 33.99%, 36.80%, and 22.65% of the total biomass, respectively. The NT region was predominantly occupied by Medium-sized Zooplankton Carnivores (MCC), accounting for 55.33% of total biomass. In the ST region, the main functional groups were MCF, SCF, and RF, with proportions of 28.03%, 30.98%, and 19.48%, respectively. In terms of biomass composition, the total biomass in ST and SA was substantially higher than in NT, which deviated from the overall biomass variation pattern.

**Figure 8:**
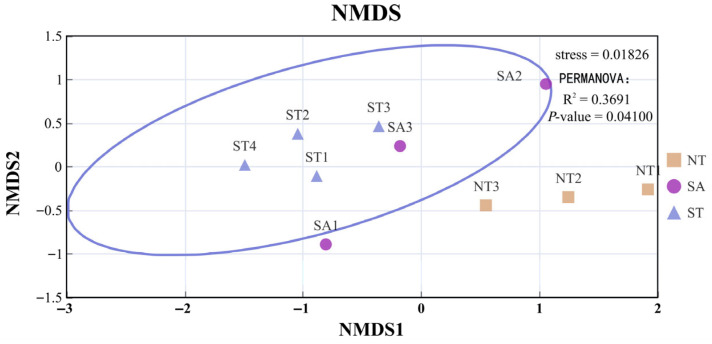
NMDS and PERMANOVE Analysis of Zooplankton Functional Groups in Three Regions.

Regarding species composition, SA and ST were relatively similar, whereas NT showed marked dissimilarity. To investigate the clustering relationships among the four sampling months, Non-metric Multidimensional Scaling (NMDS) and a cluster analysis based on the Bray–Curtis distance matrix were performed, with a confidence interval of 0.95. As shown in the figures, the overall analysis revealed significant differences among the three regions, with no obvious similarities (Figure 8).

**Figure 9:**
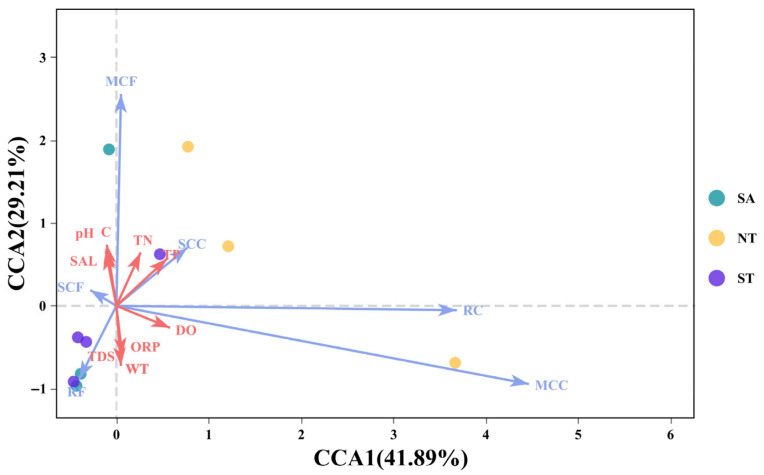
CCA of Plankton Functional Groups in Three Regions.

The results of this analysis are illustrated in Figure 9. The explanatory power of CCA1 was 41.89%, and that of CCA2 was 29.21%, with a cumulative explanatory power of 71.1% for CCA1 and CCA2 combined. The distribution of sampling sites was scattered, showing no aggregation trend. Along the CCA1 axis, total nitrogen (TN), total phosphorus (TP), dissolved oxygen (DO), total dissolved solids (TDS), and oxidation-reduction potential (ORP) were positively correlated. In contrast, other environmental factors were negatively correlated. Along the CCA2 axis, pH, salinity (SAL), conductivity (C), TN, and TP were positively correlated, whereas other environmental factors were negatively correlated.

**Figure 10:**
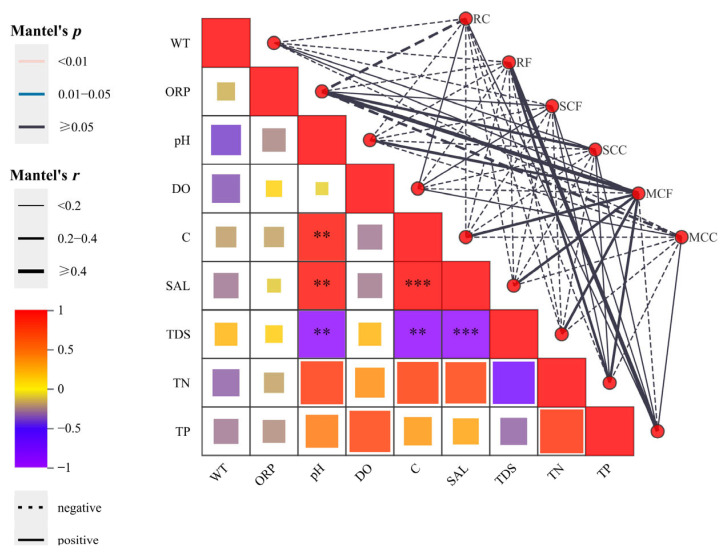
Mantel test correlation heatmap of zooplankton functional group communities and environmental variables. Note: Line thickness represents the absolute value of the correlation coefficient (Mantel’s r), line color indicates the significance level range (Mantel’s *p*), and line style (solid or dashed) denotes the direction of the correlation coefficient. ** denotes *p* < 0.01, *** denotes *p* < 0.001, and no asterisk indicates *p* > 0.05.

To evaluate the statistical significance of associations between zooplankton populations and the aquatic ecosystem, Mantel correlation analyses were implemented in the current investigation. The figure presents the relative significance of environmental variables in shaping zooplankton functional group communities. On the right side, a network diagram displays the pairwise correlations between individual environmental factors and zooplankton species, which were derived from Mantel test analyses. The line thickness corresponds to the absolute magnitude of the correlation coefficient (Mantel’s *r*), the hue of the lines reflects the spectrum of significance levels (Mantel’s *p*), and the line style (solid or dashed) signifies the directionality of the correlation coefficient (positive or negative) (Figure 10).

## 4. Discussion

### 4.1. Correlation Analysis of Ecosystem State and Water Environment Parameters in Watershed

This study investigated artificial aquatic environments in the alpine regions of Central Asia to analyze the spatial variability of aquatic environmental indicators across regions and to evaluate their impacts on zooplankton community composition, accounting for the influence of geographical barriers. In the southern Altai Mountains, salinity (SAL) showed the most fantastic range of variation. At the same time, conductivity (C), oxidation-reduction potential (ORP), total nitrogen (TN), and total phosphorus (TP) also showed significant variability, as shown in the table. These physical and nutrient indicators play crucial roles in shaping and structuring zooplankton communities [27]. High variability in nutrient substances (TN and TP) may induce significant changes in zooplankton growth rates and community structure [46]. Different zooplankton taxa have specific optimal pH ranges.

Additionally, as with nutrients, pH can indirectly affect zooplankton growth and reproduction by influencing phytoplankton standing crop [47]. As the key dietary resource for herbivorous zooplankton, fluctuations in phytoplankton biomass can impact the growth and reproductive success of zooplankton. Numerous prior investigations have established a statistically significant positive correlation between zooplankton biomass and phytoplankton cell density [37].

In contrast, artificial water bodies in the northern and southern Tianshan Mountains exhibited distinct characteristics. In the northern Tianshan Mountains, water temperature was relatively high, the variation range of dissolved DO was comparatively narrow, and nutrient indicators showed no significant differences as shown in Table 5. This may imply that the zooplankton species composition in this region is relatively stable and less susceptible to extreme environmental changes [48]. In the southern Tianshan Mountains, however, water temperature was significantly lower than in the other two regions. The substantial temperature difference resulted in marked variations in zooplankton species composition, indicating that zooplankton growth in this region is more prominently influenced by water temperature and nutrients. This finding is consistent with the research results of Stefano Simoncelli [49], who reported that zooplankton upward migration rate is closely correlated with water temperature in the migratory layer, suggesting that temperature may be a key factor regulating zooplankton activities. Global warming has accelerated snow and ice melting, which significantly impacts zooplankton by altering physical environmental conditions, food quality, and nutrient availability, thereby regulating their growth, reproduction, and community dynamics. This is highly similar to the findings of C. Laspoumaderes [50], who studied deep oligotrophic lakes in Patagonia. Their research confirmed that suspended particles carried by glacial melting form a natural light gradient, and zooplankton community turnover is driven by changes in phytoplankton carbon-to-phosphorus ratios (a proxy for food quality): regions with low C:P ratios are dominated by cladocerans (*Daphnia commutata*) with high phosphorus requirements, while regions with high C:P ratios are dominated by copepods (*Boeckella gracilipes*) with low phosphorus requirements. The present study also verified that low-temperature runoff generated by snow and ice melting, which carries large amounts of suspended particles, enters water bodies. This not only directly modifies key environmental parameters such as water turbidity and temperature but also indirectly affects the quality of zooplankton food resources by altering the carbon-to-nitrogen-to-phosphorus stoichiometric ratios of phytoplankton, ultimately influencing zooplankton growth and population maintenance [51,52].

Light-absorbing impurities in snow reduce snow surface albedo, accelerate snow and ice ablation, and alter the spatiotemporal distribution of meltwater runoff, thereby exerting cascading effects on the habitats of zooplankton [52,53]. The dynamics of this ablation process induce synergistic shifts in underwater light availability, nutrient delivery rates, and suspended particle concentrations: High-turbidity environments impair the feeding efficiency of filter-feeding zooplankton. The coupled changes in nutrients and light indirectly alter the nutritional uptake efficiency of zooplankton by shaping phytoplankton community quality. Ultimately, these alterations result in substantial differences in the dominant species composition, distribution patterns, and biomass of zooplankton communities [54,55]. Overall, variations in physical and nutrient indicators across regions have significant impacts on the ecological distribution and population dynamics of zooplankton, with geographical barriers such as the Altai and Tianshan Mountains playing a crucial role in these processes. This study investigated zooplankton community assembly in artificial water bodies near the Tianshan and Altai Mountains, focusing on regional barriers and aquatic environmental factors. The findings hold important implications for understanding the dynamic mechanisms of artificial aquatic ecosystems and zooplankton adaptation strategies in Central Asia.

### 4.2. Community Composition and Diversity of Zooplankton in the River Basin

Zooplankton play a crucial role in material cycling and energy transfer within lake ecosystem food webs and are of great significance for the stability of freshwater ecosystems [56,57]. Revealing the structural characteristics of zooplankton communities in lake systems facilitates understanding of material and energy flows in lake ecosystems [58]. The community structure, dominant species of aquatic organisms, and their relationships with aquatic environmental factors can directly reflect changes in the aquatic ecological environment [59], and their diversity indices are often used as important indicator factors for assessing water pollution levels [60]. This study explored changes in zooplankton species composition across three river basins under the influence of inter-basin aquatic environmental variation and regional barriers. A total of 85 zooplankton species were identified across 10 artificial water bodies in the three basins, including 47 rotifer, 15 copepod, and 23 cladoceran species. This is consistent with the findings of E. Bogacka-Kapusta [61] and other scholars, who demonstrated that freshwater zooplankton are mainly composed of rotifers and small crustaceans (copepods and cladocerans). Regarding zooplankton biodiversity in artificial water bodies in Xinjiang, China, the number of rotifer species is significantly higher than that of copepods and cladocerans, which aligns with survey results from other scholars on freshwater ecosystems in Central Asia [62].

Significant differences in species composition were observed among the three regions, attributed to regional barriers and variations in physical and nutrient water quality factors, with only 7 shared species across all regions. Specifically, the southern Tianshan Mountains (ST) and southern Altai Mountains (SA) shared 9 species, while ST and northern Tianshan Mountains (NT) had 10 shared species. Notably, no shared species were found between SA and NT, indicating substantial dissimilarity in species composition among the three regions. SA harbored 9 unique species, NT had 15 unique species, and ST contained 35 unique species. These survey results are consistent with historical data [62,63]. Changes in the relative and absolute biomass of zooplankton communities can affect the productivity of higher trophic levels by altering secondary production rates and energy transfer efficiency [64,65]. In this study, copepods were the most prominent zooplankton group, with nauplii absolutely dominating and almost entirely representing the Small Copepods and Cladocera Filter-feeders (SCF) functional group. Copepods, being relatively large in size, play a crucial role in aquatic ecosystems: on the one hand, they can directly influence phytoplankton abundance in water bodies; on the other hand, they serve as a natural food source for fish and other aquatic organisms [57,66]. Cladocerans and copepods feed on bacteria, algae, rotifers, and protozoa. As a food source for medium and large-sized zooplankton, rotifers transfer energy and organic matter to higher trophic levels, thereby completing the energy cycling and material transformation processes within the zooplankton community and supporting the favorable assembly of zooplankton communities. The species composition of zooplankton can indirectly reflect the characteristics of the entire river basin’s aquatic ecosystem [67]. In this study, significant differences in zooplankton density and biomass were observed among the three river basins, with NT showing substantially lower values than the other two regions. The large fluctuations in nutrient indicators (TN, TP) in NT may have affected the overall species composition. As a food source for medium and large-sized zooplankton, rotifers transfer energy and organic matter to higher trophic levels, completing energy cycling and material transformation within the zooplankton community and further supporting the favorable assembly of zooplankton communities [68].

Diversity indices are key tools for evaluating the resilience of zooplankton communities and the overall ecological health of aquatic systems [69]. Within their research, Chainho et al. categorized the Shannon–Wiener diversity index (H′) and Margalef richness index (*d*) of zooplankton communities into five distinct levels (Table 3), namely High, Good, Moderate, Low, and Poor [45,70]. Comparison with water pollution levels (Table 6) revealed that the H′ values of zooplankton across all regions were consistently lower than the d values, indicating low pollution levels. Overall, significant differences were observed in the pollution types of artificial water bodies in Xinjiang. The SA region fell between α-Mesosaprobic and Polysaprobic, and β-Mesosaprobic; the NT region was categorized as β-Mesosaprobic; the ST region was between β-Mesosaprobic and Lightly Polluted. This outcome may be attributed to differences in regional barriers and conditions, such as glacial meltwater input. These findings are consistent with research results from artificial water bodies in other regions [71].

### 4.3. Correlation Between Zooplankton Functional Groups and Water Environmental Factors

In recent years, research on changes in zooplankton community composition under different environmental conditions has gradually expanded; however, studies investigating the impacts of regional barriers on zooplankton across entire regions remain relatively scarce [72]. Generalist species exhibit high environmental adaptability and broad ecological niches. As population density increases, interspecific competition intensifies accordingly. Competition and predation among zooplankton are also important factors influencing population dynamics [73]. Functional groups occupying different ecological niches may engage in predatory relationships, leading to mutual interactions between predators and prey that collectively drive the dynamic changes in community structure [74]. Key factors affecting zooplankton growth and the distribution of zooplankton functional groups include temperature, nutrients, the top-down effects of phytoplankton, the bottom-up effects of fish predation, and interspecific competition [75]. Significant differences in zooplankton density, biomass, and community composition have been observed across different habitats [76,77]. Artificial water bodies in Xinjiang are located in alpine regions with unique geographical locations and are rarely disturbed by human activities. Regional barriers influence their hydrological environments and are closely associated with factors such as climate and glacial meltwater. Frequent water level fluctuations in some artificial water bodies result in significant variations in water depth, with the NT region experiencing the most pronounced fluctuations. This explains why zooplankton abundance in the NT region is significantly lower than in the other two regions. This finding is consistent with the results of Shen Yuying et al., who observed changes in zooplankton community structure in Poyang Lake during extreme drought events. During periods of low water levels, zooplankton species richness and abundance decreased significantly, accompanied by substantial changes in biomass [78].

Bray–Curtis analysis results revealed significant differences in the zooplankton functional groups among the three river basins in Xinjiang. Additionally, the zooplankton community composition across artificial water bodies in Xinjiang was heterogeneous, with variations in species richness and biomass [42]. Specifically, the NT region exhibited substantially lower species richness and biomass than the SA and ST regions, which are associated with temperature fluctuations, water-level changes, and significant variations in total nitrogen (TN) and total phosphorus (TP). Phytoplankton plays a crucial role in regulating the balance of aquatic ecosystems, and temperature is a key environmental factor influencing phytoplankton growth. Zooplankton functional groups show significant responses to changes in phytoplankton communities [79]. Water temperature in the ST region was significantly lower than in SA and NT, with the NT region having the highest temperature. According to Redundancy Analysis (RDA) results, temperature was negatively correlated with zooplankton functional communities. The research on phytoplankton under the barrier in Xinjiang is also involved, Temperature is an important factor affecting phytoplankton, and diatoms were the dominant group in Xinjiang, consistent with the findings of Zi Fangze et al. [79]. Diatoms have an optimal temperature range of 10–20 °C, as verified by Song Qiurong [80] in an experiment investigating the effects of temperature on benthic algal communities. Diatoms prefer low temperatures and tolerate low light conditions; some species reach their highest relative abundance at 12–18 °C, while their competitiveness declines significantly at high temperatures (>25 °C). The NT region provided more favorable conditions for phytoplankton growth. Rotifer Filter-feeders (RF) and Small Copepods and Cladocera Filter-feeders (SCF) were the dominant zooplankton functional groups in NT. As phytoplankton serves as the primary food source for these two functional groups, the substantial increase in phytoplankton abundance resulted in significantly higher zooplankton biomass and density in the NT region compared to the other two regions.

Nutrient factors, such as total nitrogen (TN) and total phosphorus (TP), also exert significant impacts on zooplankton functional groups, with TP being the most critical driver. This finding is consistent with the results of Yun Li et al. [81], who investigated the correlation between total phosphorus and zooplankton community composition. When phosphorus resources are enriched in the environment, specific zooplankton taxa gain competitive advantages through more efficient phosphorus utilization strategies, thereby inhibiting the growth and reproduction of other coexisting species [82]. The essence of this competitive pattern lies in the fact that, in resource-rich habitats, dominant zooplankton taxa expand their survival advantages by prioritizing phosphorus acquisition and optimizing phosphorus assimilation efficiency, thereby indirectly subjecting other species to phosphorus limitation. This imbalance in resource allocation not only triggers the restructuring of zooplankton community density distribution but also exerts profound impacts on the overall structure and function of freshwater ecosystems through food chain cascading effects. The formation of such interspecific competitive differences is closely related to the adaptive mechanisms of zooplankton in high-phosphorus environments that have evolved over long-term evolution. This aligns with the findings of the present study: the NT region had the highest TP values with substantial fluctuations, leading to decreased diversity and lower species biomass and density compared to the other two regions. This study provides fundamental data on changes in zooplankton species composition across regional barriers in unique artificial water bodies in high-latitude arid regions of Central Asia. As a core food source for economically important aquatic organisms such as fish, zooplankton diversity and abundance directly determine the productivity of regional aquaculture and surrounding fisheries, providing a foundation for the development of ecological fisheries and food security in arid regions. Furthermore, this research is of profound significance for maintaining the unique aquatic ecological pattern and enhancing regional ecological resilience in the high-latitude arid regions of Central Asia.

## 5. Conclusions

Surveys of aquatic environmental parameters and zooplankton community structure in 10 artificial water bodies in the southern foot of the Altai Mountains and the northern and southern slopes of the Tianshan Mountains revealed significant spatial variability across regions, with zooplankton community characteristics being closely correlated with aquatic environmental factors. The specific results are as follows: Among the physical and nutrient indicators surveyed, inter-regional variability was significant. Specifically, key environmental factors in artificial water bodies of the southern foot of the Altai Mountains (SA region) and the northern slope of the Tianshan Mountains (NT region) fluctuated sharply, with indicators such as temperature, dissolved oxygen (DO), total nitrogen (TN), and total phosphorus (TP) exhibiting substantial changes. In contrast, nutrient indicators (TN, TP) in water bodies of the southern slope of the Tianshan Mountains (ST region) showed relatively small variation ranges, indicating higher environmental stability. This inter-regional environmental heterogeneity is directly associated with regional barriers formed by the Altai and Tianshan Mountains, as well as differences in glacial meltwater recharge.

Zooplankton species identification results indicated significant differences in community composition among regions, a conclusion that was consistently verified by cluster analysis and Non-metric Multidimensional Scaling (NMDS). A total of 19 dominant zooplankton species were identified across the three river basins (SA, NT, and ST regions) and could be classified into 6 functional groups based on their ecological traits. The composition of zooplankton functional groups varied significantly across basins, with distinct regional differentiation in the types and proportions of dominant functional groups in the SA, NT, and ST regions. Apparent differences existed in zooplankton diversity and water quality indicator characteristics among the three basin.

Mountain barriers in the arid regions of Xinjiang significantly regulated zooplankton species composition, functional group differentiation, and diversity distribution by altering the spatial distribution and temporal fluctuations of water temperature, dissolved oxygen, and nutrients. As a unique hydrological factor in the region, glacial meltwater further exacerbated community differences among different basins. The above results clarify the driving mechanisms of zooplankton communities under regional barriers, providing a critical scientific basis for water resource management and environmental protection of artificial water bodies in arid Central Asia.

## Figures and Tables

**Table 1:** The longitude and latitude information of artificial water body in Xinjiang.

Reservoir	Longitude	Latitude
DL	80°46′43.261″	40°40′51.024″
SL	80°1′38.956″	40°35′56.352″
SY	79°39′48.349″	40°56′4.166″
XJZ	81°3′20.35″	40°28′0.079″
LG	83°55′45.152″	43°56′41.659″
DQG	84°40′0.630″	43°40′37.977″
MGH	85°9′27.38″	43°34′55.335″
AKDL	88°39′39.624″	47°1′34.476″
NGQ	89°7′57.417″	46°19′12.07″
KZS	89°59′21.199″	46°35′28.601″

**Table 2:** Description of zooplankton functional groups in freshwater ecosystems [35,36].

Functional Group	Size/mm	Feeding Habits
Rotifer filter feeders, RF *	-	They feed on bacteria, algae, and organic matter
Rotifer carnivora, RC *	-	They feed on protozoa, other rotifers, and small crustaceans
Rotifer predators, RP	-	They feed mainly on algae
Small copepods and cladocera filter feeders, SCF *	<0.7	They feed on bacteria, algae, organic matter, and protozoa
Small copepods and cladocera carnivora, SCC *	<0.7	They feed on rotifers, cladocera, dipteran insects (larvae of mosquitoes), and oligochaetes
Middle copepods and cladocera filter feeders, MCF *	0.7~1.5	They feed on bacteria, algae, organic matter, and protozoa
Middle copepods and cladocera carnivora, MCC *	0.7~1.5	They feed on rotifers, cladocera, dipteran insects (larvae of mosquitoes), and oligochaetes
Large copepods and cladocera filter feeders, LCF	>1.5	They feed on bacteria, algae, organic matter, and protozoa
Large copepods and cladocera carnivora, LCC	>1.5	They feed on rotifers, cladocera, dipterans (larvae of midges), and oligochaetes

Note: The functional groups assigned in this study are marked with *. Functional groups marked with ‘-’ are classified based on ‘feeding habit + reproductive type,’ and body size is not used as a classification indicator.

**Table 3:** Categorization of diversity indices and abundance metrics for zooplankton communities [45].

Classifications	Diversity Index (*H*′)	Richness Index (*d*)
High	>4.0	>4.0
Good	3.0~4.0	>4.0
Moderate	2.0~3.0	2.5~4.0
Poor	1.0~2.0	<2.5
Bad	0.0~1.0	<2.5

**Table 4:** Dominant species and dominance indices of zooplankton communities in the three regions.

*Species*	SA	NT	ST
*Brachionus plicatilis*	0.291	/	*/*
*Nauplius copepoda*	0.264	/	0.045
*Keratella quadrata*	0.264	/	*/*
*Cyclopoida larva*	0.039	0.086	0.243
*Synchaeta stylata*	0.038	/	*/*
*Daphnia galeata*	0.023	/	*/*
*Trichocerca pusilla*	/	0.257	*/*
*Asplanchna priodonta*	/	0.196	0.092
*Bosmina longirostris*	/	0.187	0.082
*Diaphanosoma brachyurum*	/	0.147	*/*
*Thermocyclops taihokuensis*	/	0.111	*/*
*Brachionus calyciflorus*	/	0.103	0.245
*Polyarthra trigla*	/	0.091	0.448
*Brachionus calyciflorus*	/	/	0.210
*Keratella valga*	/	/	0.169
*Bosmina fatalis*	/	/	0.137
*Brachionus quadridentatus*	/	/	0.096
*Bosmina coregoni*	/	/	0.087
*Keratella cochlearis*	/	/	0.075

Note “/” indicates that the species is a non-dominant species during the sampling period.

**Table 5:** Environmental factors of water bodies in the study area (mean ± standard deviation).

Environmental Factor	SA	NT	ST
WT (°C)	21.98 ± 4.11	22.56 ± 3.89	12.77 ± 4.17
pH	8.72 ± 0.22	7.72 ± 0.28	7.60 ± 0.25
SAL (‰)	0.21 ± 0.15	0.07 ± 0.03	0.08 ± 0.07
TDS (mg/L)	34.55 ± 16.33	136.07 ± 52.96	222.25 ± 164.19
C (mg/L)	416.66 ± 296.36	222.07 ± 62.29	328.35 ± 77.84
DO (mg/L)	5.22 ± 1.99	6.69 ± 1.66	5.85 ± 2.30
ORP (mg/L)	91.77 ± 53.46	91.14 ± 30.91	96.55 ± 25.95
TN (mg/L)	0.87 ± 0.32	0.58 ± 0.12	0.15 ± 0.02
TP (mg/L)	0.64 ± 0.24	0.82 ± 0.16	0.08 ± 0.07

**Table 6:** Correlation between plankton diversity index, nutrient concentrations, and water pollution types [71].

Types of Water Pollution	Diversity Index (*H*′)	Richness Index (*d*)
Oligosaprobic	>4.0	>4.0
Lightly polluted	>3.0~4.0	>3~4.0
β-Mesosaprobic	>1.0~3.0	2~3
α-Mesosaprobic to polysaprobic	0~1	0~2

## Data Availability

The data supporting this study’s findings are available from the corresponding authors upon reasonable request.
